# Determining the Impedance of an Eddy Current Probe Placed over a Defect-Free Conductive Cylinder with a Centred Circular Hole

**DOI:** 10.3390/ma19132718

**Published:** 2026-06-24

**Authors:** Grzegorz Tytko, Yike Xiang, Yao Luo

**Affiliations:** 1 Faculty of Automatic Control, Electronics and Computer Science, Silesian University of Technology, Akademicka 16, 44-100 Gliwice, Poland; 2 State Key Laboratory of Power Grid Environmental Protection, School of Electrical Engineering and Automation, Wuhan University, Wuhan 430072, China; 3 School of Electrical Engineering and Automation, Wuhan University, Wuhan 430072, China

**Keywords:** eddy current probe, coil impedance, defectoscopy, non-destructive testing, defect detection

## Abstract

The measurement of a probe impedance performed during eddy current inspections enables detection of flaws in electrically conductive materials. A correct interpretation of the measured impedance values constitutes a key aspect that determines the effectiveness of the inspections, and for this purpose, mathematical models are employed. Such models, which are becoming more and more frequently an integral part of eddy current measurement systems, enable carrying out the calculation of the probe impedance, through depicting the measurements being performed. What offer the shortest calculation time while maintaining high accuracy are analytical solutions. In this paper, to the best of the authors’ knowledge, this is the first time an analytical model of an eddy current probe placed over a small diameter cylinder containing a hole has been presented. The final formulas were obtained using the truncated region eigenfunction expansion (TREE) method, and then implemented in Matlab. The calculated values of the probe resistance and reactance were compared with the measurement results obtained for cylinders with a through defect. The tests were conducted on components made of several conductive materials with different geometric dimensions. The measurement error in all of the tests was small, i.e., it did not exceed 3% across the entire frequency range. The proposed solution can be used in defectoscopy for eddy current testing of tubes, pucks, washers, and any cylindrical elements.

## 1. Introduction

**Figure 1:**
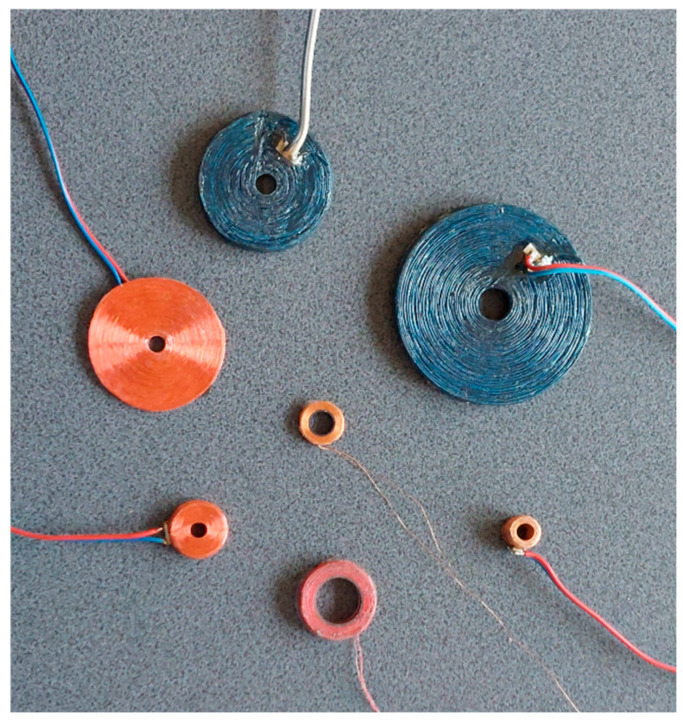
The air-core coils.

Eddy current testing (ECT) is one of the most commonly used non-destructive methods for the evaluation of electrically conductive materials. The dynamic development of this measurement technique results from, among other things, the significant increase in the computing power of processors, and new possibilities for computer simulations. Eddy current inspections are performed by qualified personnel who examine metal components using a probe. Depending on the type of the tested object, different probes are employed, i.e., probes consisting of a single coil [1,2,3], operating in a transmitter-receiver configuration [4,5,6,7,8], or containing a system of multiple coils [9,10,11]. The simplest probe, which is powered by alternating current, takes the form of a coil wound on a carcass (Figure 1). Bringing the probe close to the surface of the tested material results in generating eddy currents in the material. Any changes in the structure and electromagnetic properties of the test object disrupt the flow of eddy currents, causing a change in the probe’s impedance. And thus, by measuring impedance values, it is possible to detect defects [12,13,14,15] and determine certain parameters of the tested element, such as thickness [16,17,18,19], radius [20,21], or electrical conductivity [22,23].

What is a key aspect of the inspections that are carried out is that they determine that the correct assessment of the technical condition of the tested product is the correct interpretation of the measured probe impedance values. For this purpose, mathematical models, which enable impedance calculations for various input data, are used. The obtained results can be used before the inspection to determine predicted values of the probe impedance, and after the inspection, to interpret the measurement results. The most commonly used models in eddy current testing are numerical models developed with the employment of the finite element method (FEM) [24,25,26] or boundary element method (BEM) [27,28,29]. The creation of an appropriate numerical model with computing software such as Comsol Multiphyscics or Ansys Maxwell is relatively simple and fast. Unfortunately, models based on mesh methods require a relatively long time for computation. This constitutes a significant limitation, especially in applications that require thousands of computational iterations. Therefore, for many years, a gradual development of analytical modelling has been taking place, resulting in achieving solutions to more and more complex eddy current problems. Analytical models are very fast, and the final formulas presented in closed form allow their implementation in any programming language or computing environment. The short time required to obtain final results is mainly due to the use of preliminary calculations. Thanks to this, all mathematical operations are performed in the first iteration, whereas the subsequent iterations only deal with the operations for which the input data have changed. Unfortunately, the derivation of an analytical model and then its numerical implementation is a difficult and time-consuming process. Despite their efforts, scientists still have not been able to develop analytical solutions for numerous eddy current problems.

The dynamic development of analytical modelling in the field of eddy current testing occurred after the development of the truncated region eigenfunction expansion method (TREE) [30]. Initially, models obtained in this way allowed calculations for materials containing no defects [31], which significantly limited their practical usefulness. Therefore, the derivation of a model of a coil placed over a conducting plate with a hole was an important step [32]. The development of effective root-finding algorithms [33,34,35] made it possible to significantly expand the scope of the previously considered geometries. The tested material no longer had to have geometric dimensions as large as the solution domain, which constituted one of the most significant limitations of analytical models. The models derived in subsequent works were intended for testing cylindrical materials with small diameters, for air-core coils [36], I-core coils [37], and pot-core coils [38]. Although this made it possible to perform calculations for elements with small geometric dimensions, all of these solutions had very limited usefulness because of the fact that the presence of defects in conductive material was not taken into account. This limitation resulted from the complex nature of the mathematical expressions and the occurrence of numerous numerical problems. The complexity of this problem was the reason why, despite its high usefulness, it was not possible to develop an appropriate analytical model for this case. In this paper, it is the first time a solution to this problem has been presented.

The novelty of this paper consists in the concept of the analytical model of a probe placed over material in the shape of a cylinder with a small diameter, containing a defect. The presented solution constitutes a complement to the eddy current system based on the measurement of the probe impedance. It enables computational simulation of the measurements that were carried out, verification of their correctness, and correct interpretation of the obtained measurement data. In the first step, using the TREE method, the solution domain was divided into regions with different electromagnetic properties, for which expressions for the magnetic vector potential were determined. Subsequently, boundary conditions and conditions of magnetic field continuity were applied at the interfaces between the regions and subregions. The solving of the resulting system of equations made it possible to determine the final formula for the probe impedance. This expression was presented in closed form, thus enabling its implementation in Matlab R2025b. Numerical problems associated with determining the eigenvalues were overcome with the employment of the authors’ approach based on the 1D-FEM. Calculations of the probe impedance were performed for three flat cylinders with a through hole. These cylinders had different geometric dimensions and were made of brass, bronze, and aluminium. The comparison between the results of the impedance calculation and the measurement results showed a very good agreement. In all cases, for both resistance and reactance, the error did not exceed 3%. The presented analytical solution enables rapid calculations of probe impedance, while taking into account the presence of a through hole, of any depth and diameter, in the material. The developed analytical model can find practical applications in defectoscopy for eddy current testing of various elements, such as pipes, cylinders, rings, and washers.

## 2. Materials and Methods

### 2.1. Analytical Model

**Figure 2:**
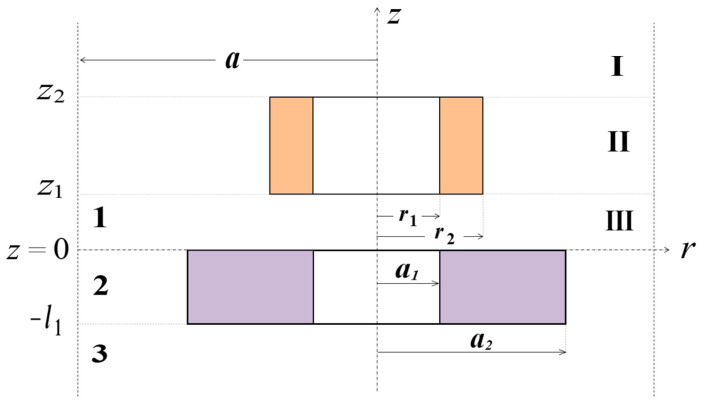
Cross-section of an air-core coil located above a conductive cylinder with a hole, for which two types of regions are defined. Region I is above the coil (*z* ≥ *z*_2_), region II comprises the coil (*z*_2_ ≥ *z* ≥ *z*_1_), and region III is below the coil (*z* ≤ *z*_1_). Region 1 is above the tested material (*z* ≥ 0), region 2 is a conducting cylinder (0 ≥ *z* ≥ −*l*_1_), and region 3 is below the tested material (*z* ≤ −*l*_1_).

The cross-section of an air-core coil placed over a conductive cylinder with a hole is shown in Figure 2. The coil with height *z*_2_-*z*_1_ and width *r*_2_-*r*_1_ was considered in a cylindrical coordinate system for the solution domain limited in the radial direction *r* to the value of parameter *a*. The coil is placed at a distance *z*_1_ from the surface of the cylinder with thickness *l*_1_ and electrical conductivity *σ*. The outer radius of the cylinder was designated as *a*_2_, and the radius of the hole as *a*_1_. In the first step, the solution domain limited in the *z* direction was divided into three parts. Region I is located above the coil (*z* ≥ *z*_2_), region II comprises the coil (*z*_2_ ≥ *z* ≥ *z*_1_), and region III is below the coil (*z* ≤ *z*_1_). For each of such defined regions, an expression for the magnetic vector potential was formulated [30].

(1) $$A_{I}\left(r,z\right)=J_{1}^{T}\left(r\right)e^{-zA}D_{1},$$

(2) $$A_{II}\left(r,z\right)=J_{1}^{T}\left(r\right)[e^{zA}C_{2}+e^{-zA}D_{2}+V],$$

(3) $$A_{III}\left(r,z\right)=J_{1}^{T}\left(r\right)e^{zA}C^{\left(e\right)},$$

where *J*_n_(*x*) is the Bessel function of the first kind of order *n*, $J_{1}^{T}\left(r\right)$ is a row vector, and **V** is the column vector with elements

(4) $$V_{i}=\frac{2\mu_{0}}{{{[\alpha }_{i}aJ_{0}\left(\alpha_{i}a\right)]}^{2}}\kappa \left(\alpha_{i},r_{1},r_{2}\right).$$

The eigenvalues *α_i_* are the positive roots of *J*_1_(*α_i_ a*) = 0, *μ*_0_ is the vacuum magnetic permeability, and

(5) $$\kappa \left(\alpha_{i},r_{1},r_{2}\right)=\int_{r_{1}}^{r_{2}}rJ_{1}\left(\alpha_{i}\;r\right)dr.$$

Equations (4) and (5) define the elements of the column vector **V**, which is obtained from the source expansion of the Poisson equation in cylindrical coordinates. After separation of variables, the source term is expanded in terms of the radial eigenfunctions $J_{1}(\alpha_{i}r)$, where the eigenvalues $\alpha_{i}$ are the positive roots satisfying the truncated boundary condition $J_{1}(\alpha_{i}a)=0$. Using the orthogonality properties of the Bessel eigenfunctions, the source projection coefficient can be written as

(6) $$\psi (\alpha_{i})=\frac{2}{a^{2}J_{0}^{2}(\alpha_{i}a)}\int_{r_{1}}^{r_{2}}rJ_{1}(\alpha_{i}r)\;dr.$$

The quantity $\kappa (\alpha_{i},r_{1},r_{2})$ is introduced as a compact representation of the integral term. The normalisation factor $a^{2}J_{0}^{2}(\alpha_{i}a)$ arises from the orthogonality relation of the Bessel eigenfunctions in the truncated radial domain. Consequently, the vector element

(7) $$V_{i}=\frac{\mu_{0}\psi (\alpha_{i})}{\alpha_{i}^{2}}$$

is obtained from the source projection and the corresponding eigenvalue operator of the radial equation.

Other matrices and vectors in (1)–(3) are as follows:

(8) $$A=\left[\begin{array}{ccc} \alpha_{1} & 0 & \ldots \\ 0 & \alpha_{2} & \ldots \\ \vdots & \vdots & \ddots \end{array}\right],e^{Az}=\left[\begin{array}{ccc} e^{\alpha_{1}z} & 0 & \ldots \\ 0 & e^{\alpha_{2}z} & \ldots \\ \vdots & \vdots & \ddots \end{array}\right],J_{1}\left(r\right)=\left[\begin{array}{c} J_{1}(\alpha_{1}r)\\ J_{1}(\alpha_{2}r)\\ \vdots \end{array}\right],$$

and **D**_1_, **D**_2_, **C**_2_, **C**^(e)^ are coefficients to be determined. Applying the interface continuity conditions of *B_r_* and *H_z_* at *z* = *z*_1_ and *z* = *z*_2_ to the magnetic vector potential expansions in regions I–III makes it possible to obtain Equation (9). Substituting the separated-variable solutions into the continuity conditions of the tangential magnetic field and normal magnetic flux density, a matrix system for the unknown coefficient vectors is obtained. Solving this system yields the following expression for **C**^(e)^.

(9) $$C^{\left(e\right)}=\frac{1}{2}\left(e^{-Az_{1}}-e^{-Az_{2}}\right)V.$$

The determination of the coefficient **C**^(e)^ allows solving the system of interface equation. For this purpose, the solution domain of the problem shown in Figure 2 was divided into three regions. Region 1 is located above the surface of the tested material (*z* ≥ 0), region 2 is a conducting cylinder (0 ≥ *z* ≥ −*l*_1_), and region 3 is the space below the lower edge of the tested material (*z* ≤ −*l*_1_). The expressions for the magnetic vector potential of these regions were obtained by using the separation of variables, in a similar manner to (1)–(3).

(10) $$A_{1}\left(r,z\right)=J_{1}^{T}\left(r\right)[e^{zA}C^{\left(e\right)}+e^{-zA}D_{1}],$$

(11) $$A_{2}\left(r,z\right)=F^{T}\left(r\right)[e^{zP}C_{2}+e^{-zP}D_{2}],$$

(12) $$A_{3}\left(r,z\right)=J_{1}^{T}\left(r\right)e^{zA}C_{3},$$

where

(13) $$P=\left[\begin{array}{ccc} p_{1} & 0 & \ldots \\ 0 & p_{2} & \ldots \\ \vdots & \vdots & \ddots \end{array}\right],F\left(r\right)=\left[\begin{array}{c} f_{1}(p_{1}r)\\ f_{2}(p_{2}r)\\ \vdots \end{array}\right],$$

and the superscript **T** denotes transposition, which means that $J_{1}^{T}\left(r\right)$ and $F^{T}\left(r\right)$ are row vectors.

Under consideration of the interface conditions of $B_{r}=-\partial A/\partial z$ and $H_{z}=1/r\;[\partial \left(rA\right)/\partial r]$ at *z* = 0 and *z* = −*l*_1_, the following equations for the coefficients **D**_1_, **C**_2_, **D**_2_ and **C**_3_ can be written.

(14) $$A_{1}\left(r,0\right)=A_{2}\left(r,0\right)$$

(15) $$-{\left\{\frac{\partial A_{1}}{\partial z}\right\}}_{z=0}=-{\left\{\frac{\partial A_{2}}{\partial z}\right\}}_{z=0}$$

(16) $$A_{2}\left(r,-l_{1}\right)=A_{3}\left(r,-l_{1}\right)$$

(17) $$-{\left\{\frac{\partial A_{2}}{\partial z}\right\}}_{z=-l_{1}}=-{\left\{\frac{\partial A_{3}}{\partial z}\right\}}_{z=-l_{1}}$$

By imposing interface conditions between adjacent regions together with (10)–(12), we obtain

(18) $$J_{1}^{T}\left(r\right)\left[C^{\left(e\right)}+D_{1}\right]=F^{T}\left(r\right)\left[C_{2}+D_{2}\right]$$

(19) $$J_{1}^{T}\left(r\right)A\left[C^{\left(e\right)}-D_{1}\right]=F^{T}\left(r\right)P\left[C_{2}-D_{2}\right]$$

(20) $$F^{T}\left(r\right)\left[e^{l_{1}P}C_{2}+e^{-l_{1}P}D_{2}\right]=J_{1}^{T}\left(r\right)e^{l_{1}A}C_{3}$$

(21) $$F^{T}\left(r\right)P\left[e^{l_{1}P}C_{2}-e^{-l_{1}P}D_{2}\right]=J_{1}^{T}\left(r\right)Ae^{l_{1}A}C_{3}$$

Finally, by left-multiplying the matrix equations by $rJ_{1}\left(r\right)$ or $rF\left(r\right)$ and integrating from 0 to *a*, the interface equations have been formulated.

(22) $$S\left[C^{\left(e\right)}+D_{1}\right]=T_{1}\left[C_{2}+D_{2}\right],$$

(23) $$T_{1}^{T}A\left[C^{\left(e\right)}-D_{1}\right]=P\left[C_{2}-D_{2}\right],$$

(24) $$e^{l_{1}P}C_{2}+e^{{-l}_{1}P}D_{2}=T_{1}^{T}e^{l_{1}A}C_{3},$$

(25) $$T_{1}P[e^{l_{1}P}C_{2}-e^{{-l}_{1}P}D_{2}]=SAe^{l_{1}A}C_{3},$$

where

(26) $$S=\int_{0}^{a}rJ_{1}\left(r\right)J_{1}^{T}\left(r\right)dr=\frac{a^{2}}{2}\left[\begin{array}{ccc} J_{0}^{2}\left(\alpha_{1}a\right) & 0 & \ldots \\ 0 & J_{0}^{2}\left(\alpha_{2}a\right) & \ldots \\ \vdots & \vdots & \ddots \end{array}\right],$$

and

(27) $$T_{1}=\int_{0}^{a}rJ_{1}\left(r\right)F^{T}\left(r\right)dr$$

is evaluated by Clenshaw–Curtis quadrature, which is preferable to the Gauss quadrature for the oscillatory integrands. From Equations (22)–(25), we arrive at

(28) $$\left[\begin{array}{cccc} S & -{Τ}_{1} & -{Τ}_{1} & 0\\ -T_{1}^{T}A & -P & P & 0\\ 0 & e^{l_{1}P} & e^{-l_{1}P} & -T_{1}^{T}e^{l_{1}A}\\ 0 & T_{1}Pe^{l_{1}P} & -T_{1}Pe^{-l_{1}P} & -SAe^{l_{1}A} \end{array}\right]\left[\begin{array}{c} D_{1}\\ C_{2}\\ D_{2}\\ C_{3} \end{array}\right]=\left[\begin{array}{c} -SC^{\left(e\right)}\\ -T_{1}AC^{\left(e\right)}\\ 0\\ 0 \end{array}\right]$$

By solving (28), we can obtain **D**_1_, **C**_2_, **D**_2_ and **C**_3_. Considering that the inductance is independent of the excitation current in the wire, the current density *J* on the right-hand side of (29) is omitted by setting *J* = 1 A/m^2^ during the solution process. Since the current density of the uniformly wound coil is related to the excitation current by *J* = *NI/S*, where *N* is the number of turns and *S* is the coil cross-sectional area, the probe impedance change, caused by the presence of the testing material, can be expressed as

(29) $$\Delta Z=\frac{i\omega }{I^{2}}\int_{V}\Delta A_{1\;}\cdot JdV=\frac{2\pi i\omega N^{2}}{{\left(r_{2}-r_{1}\right)}^{2}{\left(z_{2}-z_{1}\right)}^{2}}\sum_{j=1}^{\infty }\kappa (\alpha_{j},r_{1},r_{2})\frac{e^{-\alpha_{j}z_{2}}-e^{-\alpha_{j}z_{1}}}{\alpha_{j}}D_{1,j}$$

where *ω* is an angular frequency.

### 2.2. Numerical Implementation

The source code enabling the calculation of the probe impedance changes was written in Matlab R2025b, according to expression (29). The Bessel functions *J*_n_(*x*) and *Y*_n_(*x*) were implemented using the besselj() and bessely() procedures. The integral (5), containing the product of the Bessel function and its argument, was expanded into the following series, which required merely 20 terms.

(30) $$\kappa \left(\alpha_{i},r_{1},r_{2}\right)=\frac{1}{\alpha_{i}}\left[r_{1}J_{0}\left(\alpha_{i}r_{1}\right)-r_{2}J_{0}\left(\alpha_{i}r_{2}\right)\right]+\frac{2}{\alpha_{i}^{2}}\sum_{n=0}^{\infty }\left[J_{2n+1}\left(\alpha_{i}r_{2}\right)-J_{2n+1}\left(\alpha_{i}r_{1}\right)\right]$$

The most challenging issue in numerical implementation is the correct calculation of the eigenvalues. What must be used for this purpose is a reliable algorithm, as omitting even a single eigenvalue usually results in incorrect results. The eigenvalues *α_i_* of the regions which do not comprise any conductive material are real numbers. For the purpose of this paper, they were calculated by the determination of the successive roots of the equation *J*_1_(*α_i_ a*) = 0 using the Newton–Raphson method. In the case of region 2, which contains a conducting cylinder with a hole, the eigenvalues *p* are complex numbers. Due to the fact that region 2 consists of three subregions, the function enabling the calculation of the eigenvalues takes on a complex form and is characterised by significant oscillations. For these reasons, it is very easy to omit the zeros of this function. This paper discusses two types of approach to determining complex eigenvalues for this type of geometry.

In the traditional TREE method, the vector potential in region 2 is

(31) $$A_{2}\left(r,z\right)=F^{T}\left(r\right)\left[e^{zP}C_{2}+e^{-zP}D_{2}\right]$$

where

(32) $$F^{T}\left(r\right)=\left\{\begin{array}{c} J_{1}^{T}\left(pr\right),\\ J_{1}^{T}\left(qr\right)B_{1}+Y_{1}^{T}\left(qr\right)B_{2},\\ J_{1}^{T}\left(pr\right)B_{3}+Y_{1}^{T}\left(pr\right)B_{4}, \end{array}\right.\begin{array}{c} \;0\leq r\leq a_{\;1}\\ \;a_{1}\leq r\leq a_{2}\\ a_{2}\leq r\leq a \end{array}$$

(33) $$J_{1}\left(pr\right)=\left[\begin{array}{c} J_{1}\left(p_{1}r\right)\\ J_{1}\left(p_{2}r\right)\\ \vdots \end{array}\right],\;Y_{1}\left(pr\right)=\left[\begin{array}{c} Y_{1}\left(p_{1}r\right)\\ Y_{1}\left(p_{2}r\right)\\ \vdots \end{array}\right].$$

Determining the coefficients **B**_1_, **B**_2_, **B**_3_, **B**_4_ requires applying the continuity conditions of $B_{r}=-\partial A/\partial z$ and $H_{z}=1/r\;[\partial \left(rA\right)/\partial r]$ at *r* = *a*_1_ and *r* = *a*_2_.

(34) $$A_{2\;(hole)}\left(a_{1},z\right)=A_{2\;(cylinder)}\left(a_{1},z\right)$$

(35) $$\frac{1}{r}{\left\{\frac{\partial \left(rA_{2\;(hole)}\right)}{\partial r}\right\}}_{r=a_{1}}=\frac{1}{r}{\left\{\frac{\partial \left(rA_{2\;(cylinder)}\right)}{\partial r}\right\}}_{r=a_{1}}$$

(36) $$A_{2\;(cylinder)}\left(a_{2},z\right)=A_{2\;(air)}\left(a_{2},z\right)$$

(37) $$\frac{1}{r}{\left\{\frac{\partial \left(rA_{2\;(cylinder)}\right)}{\partial r}\right\}}_{r=a_{2}}=\frac{1}{r}{\left\{\frac{\partial \left(rA_{2\;(air)}\right)}{\partial r}\right\}}_{r=a_{2}}$$

The resulting interface equations can be written as follows:

(38) $$J_{1}^{T}\left(pa_{1}\right)=J_{1}^{T}\left(qa_{1}\right)B_{1}+Y_{1}^{T}\left(qa_{1}\right)B_{2}$$

(39) $$PJ_{0}^{T}\left(pa_{1}\right)=QJ_{0}^{T}\left(qa_{1}\right)B_{1}+QY_{0}^{T}\left(qa_{1}\right)B_{2}$$

(40) $$J_{1}^{T}\left(qa_{2}\right)B_{1}+Y_{1}^{T}\left(qa_{2}\right)B_{2}=J_{1}^{T}\left(pa_{2}\right)B_{3}+Y_{1}^{T}\left(pa_{2}\right)B_{4}$$

(41) $$QJ_{0}^{T}\left(qa_{2}\right)B_{1}+QY_{0}^{T}\left(qa_{2}\right)B_{2}=PJ_{1}^{T}\left(pa_{2}\right)B_{3}+PY_{1}^{T}\left(pa_{2}\right)B_{4}$$

Solving this system of equations allows us to derive formulas for the elements of the diagonal matrices **B**_1_, **B**_2_, **B**_3_, **B**_4_.

(42) $$B_{1}=\frac{1}{2}a_{1}\pi \left(qJ_{1}\left(pa_{1}\right)Y_{0}\left(qa_{1}\right)-pJ_{0}\left(pa_{1}\right)Y_{1}\left(qa_{1}\right)\right)$$

(43) $$B_{2}=\frac{1}{2}a_{1}\pi \left(-qJ_{0}\left(qa_{1}\right)J_{1}\left(pa_{1}\right)+pJ_{0}\left(pa_{1}\right)J_{1}\left(qa_{1}\right)\right)$$

(44) $$\begin{array}{c} B_{3}=\frac{1}{4}a_{1}a_{2}\pi^{2}\left\{qJ_{1}\left(pa_{1}\right)\left[pJ_{1}\left(qa_{2}\right)Y_{0}\left(pa_{2}\right)Y_{0}\left(qa_{1}\right)\right.+q\left(J_{0}\left(qa_{1}\right)Y_{0}\left(qa_{2}\right)-J_{0}\left(qa_{2}\right)Y_{0}\left(qa_{1}\right)\right)Y_{1}\left(pa_{2}\right)\right.\\ \left.-pJ_{0}\left(qa_{1}\right)Y_{0}\left(pa_{2}\right)Y_{1}\left(qa_{2}\right)\right]+pJ_{0}\left(pa_{1}\right)\left[\left(qJ_{0}\left(qa_{2}\right)Y_{1}\left(pa_{2}\right)-pJ_{1}\left(qa_{2}\right)Y_{0}\left(pa_{2}\right)\right)\right.Y_{1}\left(qa_{1}\right)\\ \left.\left.+J_{1}\left(qa_{1}\right)\left(pY_{0}\left(pa_{2}\right)Y_{1}\left(qa_{2}\right)-qY_{0}\left(qa_{2}\right)Y_{1}\left(pa_{2}\right)\right)\right]\right\}, \end{array}$$

(45) $$\begin{array}{c} B_{4}=\frac{1}{4}a_{1}a_{2}\pi^{2}\left\{qJ_{1}\left(pa_{2}\right)\left(pJ_{0}\left(pa_{1}\right)J_{1}\left(qa_{1}\right)-qJ_{0}\left(qa_{1}\right)J_{1}\left(pa_{1}\right)\right)Y_{0}\left(qa_{2}\right)\right.\\ +qJ_{0}\left(qa_{2}\right)J_{1}\left(pa_{2}\right)\left(qJ_{1}\left(pa_{1}\right)Y_{0}\left(qa_{1}\right)-pJ_{0}\left(pa_{1}\right)Y_{1}\left(qa_{1}\right)\right)\\ +pJ_{0}\left(pa_{2}\right)\left[qJ_{1}\left(pa_{1}\right)\left(J_{0}\left(qa_{1}\right)Y_{1}\left(qa_{2}\right)-J_{1}\left(qa_{2}\right)Y_{0}\left(qa_{1}\right)\right)\right.\\ \left.\left.+pJ_{0}\left(pa_{1}\right)\left(J_{1}\left(qa_{2}\right)Y_{1}\left(qa_{1}\right)-J_{1}\left(qa_{1}\right)Y_{1}\left(qa_{2}\right)\right)\right]\right\}, \end{array}$$

(46) $$k=\sqrt{i\omega \sigma \mu_{0}}=\sqrt{p^{2}-q^{2}}.$$

Through the application of the conditions of the magnetic field continuity for the domain truncation *r* = *a*, the eigenfunction was obtained in the following form:

(47) $$\eta_{1}\left(p\right)=B_{3}J_{1}\left(pa\right)+B_{4}Y_{1}\left(pa\right)$$

The eigenvalues correspond to the roots of *η*_1_(*p*) = 0. Subsequently, according to the argument principle in complex analysis, the eigenvalues enclosed by the contour *Γ* in the complex plane may be extracted using the contour integral expression

(48) $$p=\frac{1}{2\pi i}\oint_{\Gamma }p\frac{\eta_{1}^{\prime }\left(p\right)}{\eta_{1}\left(p\right)}dp$$

A different approach was used in this work for the calculations performed using the 1D-FEM. This procedure follows the normalised eigenfunction expansion and numerical integration strategy developed in [39,40], where the complete implementation details are provided. For a balance between computational efficiency and numerical accuracy, the radial eigenfunctions are discretised using a one-dimensional finite element interpolation based on cubic Lagrange polynomials. On each radial element, the eigenfunction is approximated by a cubic interpolation defined on a reference coordinate −1≤ *ξ* ≤1.

(49) $$\chi \left(r\right)=\sum_{j=0}^{3}\chi_{j}N_{j}\left(\xi \right)$$

where *N_j_*(*ξ*) are the cubic Lagrange shape functions associated with the Gauss–Lobatto nodes, and *χ_j_* are the nodal interpolation coefficients.

After substituting (45) into the governing Sturm–Liouville equation and applying the Galerkin weighted-residual procedure, the weak form of the eigenvalue problem can be obtained. By assembling the element-level contributions over all radial finite elements, the generalised matrix eigenvalue equation is derived as

(50) $$KU=WUP^{2}$$

with **K** and **W** denoting the stiffness and mass matrices respectively, **U** is the eigenvector containing the nodal unknowns, and **P** is the corresponding eigenvalue. The corresponding matrix entries are obtained by assembling element-level contributions involving the first-order derivatives of the shape functions and the effective radial potential term.

To ensure numerical stability for high-order and complex eigenvalues, the eigenfunctions are normalised and all oscillatory integrals are evaluated using the Clenshaw–Curtis quadrature scheme. This quadrature is particularly suitable for highly oscillatory integrands and has been shown to effectively suppress numerical overflow in multi-subdomain eigenvalue problems.

## 3. Results

**Figure 3:**
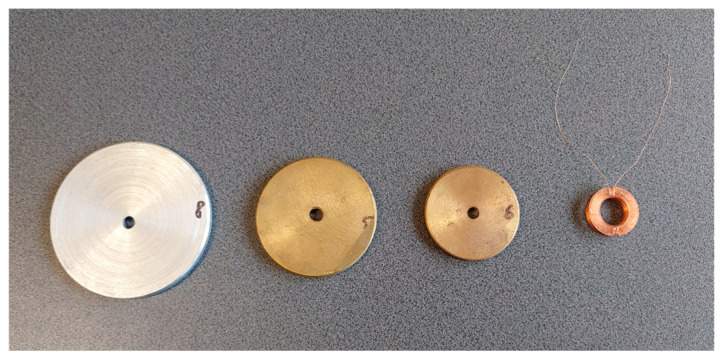
Air-core coil and conductive samples used in experiments.

Verification of the developed analytical model was performed by carrying out the measurements of the probe’s impedance components. For this purpose, an air-core coil, the parameters of which are given in Table 1, containing 1550 turns, was wound using copper wire (Figure 3). The probe’s power supply cables were connected to a Keysight E4980A precision LCR metre. The measurements of the probe’s impedance components were performed for 100 frequency values in the range from 500 Hz to 15 kHz, with an accuracy of ±0.05%. The first step was to measure the resistance *R*_0_ and reactance *X*_0_ of the probe, in the vicinity of which no conductive materials were placed. In the next step, the measurements of the resistance *R* and reactance *X* of the probe placed above a conductive cylinder with a hole were taken. Three cylinders of different geometric dimensions were tested, made of bronze, brass, and aluminium, the parameters of which are presented in Table 2.

**Figure 4:**
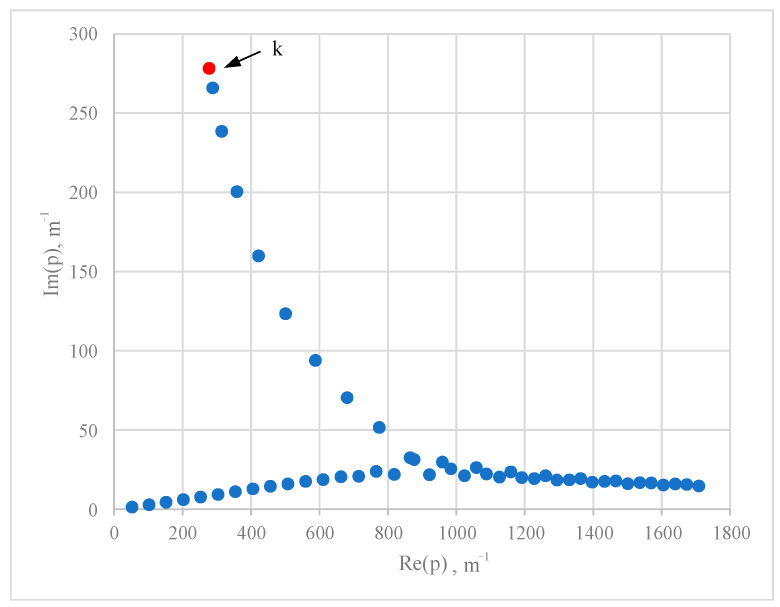
The distribution of *p* in complex plane when *f* = 1 kHz.

Calculations of the changes in the probe’s impedance Δ*Z* = *Z* − *Z*_0_ were performed using the expression (29), which was implemented in Matlab. The domain was confined to a value *a* = 10 × *r*_2_, and the distance between the coil and the tested cylinder (lift-off) was set to *z*_1_ = 0.01 mm. The eigenvalues *p* were first calculated with the TREE approach. Unfortunately, for the contour integral method, the overflow issue has occurred, resulting in inaccurate evaluations of the complex eigenvalues *p*, even with the eigenfunctions in the down sequence. The employment of 1D-FEM in the subsequent step resulted in the successful determination of all eigenvalues *p*. The calculations were based on a set of 50 values, the distribution of which is presented in Figure 4. The first 20 eigenvalues *p* determined for a frequency of *f* = 1 kHz are shown in Table 3. The coefficient *k* represents the wavenumber in the conducting material and its value for the frequency *f* = 1 kHz is (278.10 + *i* 278.10) m^−1^.

**Figure 5:**
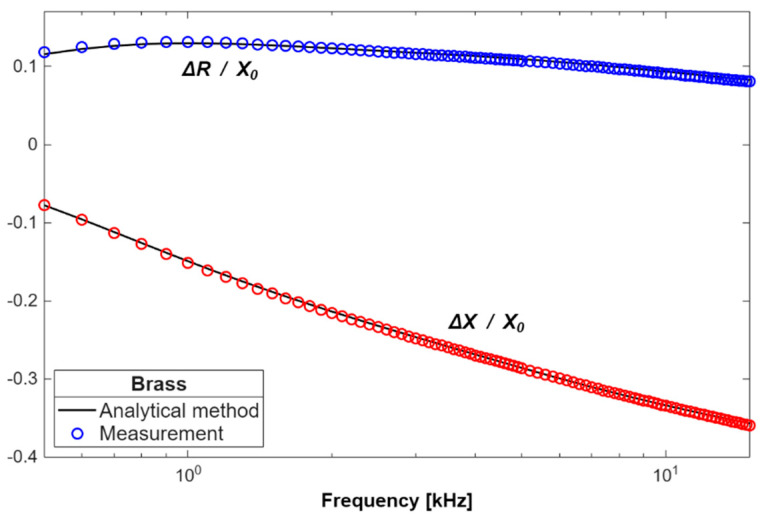
Normalised changes in the probe’s impedance components for the cylinder made of brass.

**Figure 6:**
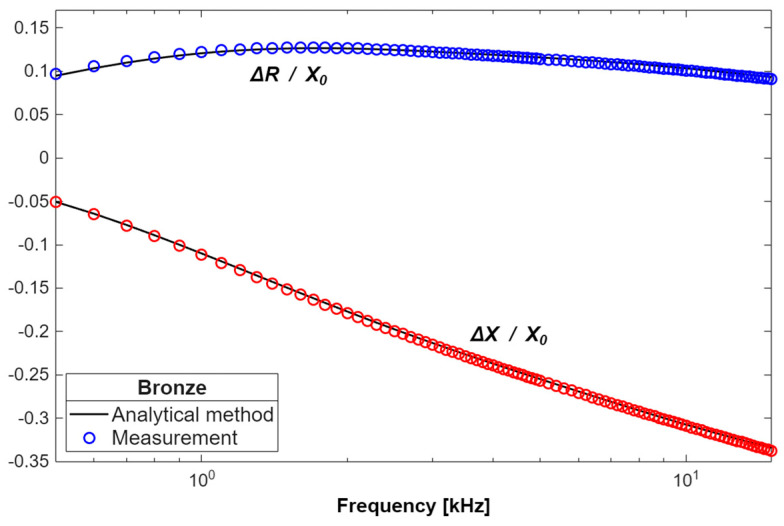
Normalised changes in the probe’s impedance components for the cylinder made of bronze.

**Figure 7:**
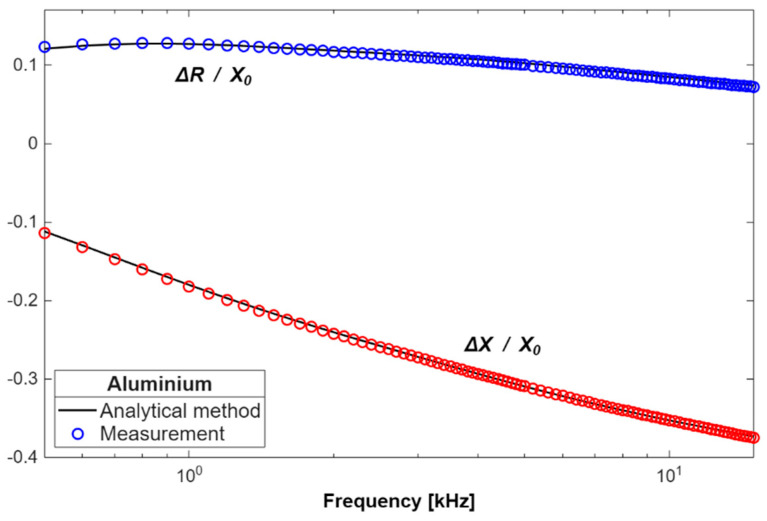
Normalised changes in the probe’s impedance components for the cylinder made of aluminium.

The changes in the impedance components obtained through the measurements and calculations were normalised with respect to the reactance *X*_0,_ and presented as a function of frequency in Figure 5, Figure 6 and Figure 7. The numerical values of the resistance Δ*R* = *R* − *R*_0_ and reactance Δ*X* = *X* − *X*_0_ obtained for the various cylinders are also compared in Table 4 and Table 5. For this purpose, it was necessary to define the errors in determining the changes in the resistance and in the reactance, that is, *dR* and *dX*, respectively.

(51) $$dR=100\%\cdot \frac{\Delta R_{analytical}-\Delta R_{measurement}}{\Delta R_{measurement}}$$

(52) $$dX=100\%\cdot \frac{\Delta X_{analytical}-\Delta X_{measurement}}{\Delta X_{measurement}}$$

## 4. Discussion

The implementation of the analytical model in Matlab enabled a rapid calculation of the changes in the impedance of the probe placed over a conductive cylinder with a hole. Matrix operations and the availability of numerous routines significantly simplified the development of the source code, which meant that the access to external libraries was not necessary. What constituted the greatest challenge was the determination of the complete set of 50 complex eigenvalues *p*. In the first step, the simplest case, i.e., where the radius of the hole is very small, was dealt with. Then, the number of eigenvalues *p* was being gradually increased, which resulted in achieving increasingly higher accuracy. It was determined that the further increasing of the number of eigenvalues above 50 no longer reduces the calculation error. It should be remembered that the greater the number of eigenvalues *p*, the more calculations are to be performed and the longer the time to obtain the final results; therefore, it is aimed at using the smallest possible number of eigenvalues that would be sufficient to ensure the required accuracy. Initially, the classic TREE approach with contour integration was employed (48). However, it turned out that the calculation of all eigenvalues *p* with this method was infeasible. The first obstacle was the occurrence of overflow issue resulting from the oscillatory nature of the integrand function. Consequently, both the determination of the *p* value and also the determination of the number of eigenvalues in the contour is difficult. In order to solve the problem, an adaptive algorithm for the partitioning of the solution domain should be used, which requires a larger number of time-consuming integrations. Another difficulty was the phenomenon of zero skipping, which occurs due to the very steep slope of the integral function’s characteristic. In this case, the zero must be calculated with a very high accuracy. Otherwise, the given value is not treated as a zero and thus it is omitted. Verification whether the eigenvalue has been determined correctly is also difficult, since even a small loss of precision in calculating the zero results in the function not assuming a zero value for that point. For these reasons, the calculation of the eigenvalues *p* was performed using the 1D-FEM.

The time of a single iteration of calculating the change in the probe impedance in Matlab was 0.84 s. This result was compared to the time of calculations with the employment of the finite element method. The Comsol Multiphysics 6.0 program was used to develop a numerical model, whose solution domain was divided into a mesh composed of triangular elements. By changing the mesh parameters, it was observed that results with a small error may be obtained when the number of triangular elements is no lower than 10,000. In the next step, the average time taken to determine the change in the probe impedance was measured—it was approximately 7 s, which is eight times longer than in the case of calculations using the analytical model.

The calculations and measurements of impedance were performed for frequencies from 500 Hz to 15 kHz. Such a range of the probe’s operating frequency was chosen in order to achieve an eddy current penetration depth at least as great as the thickness of the cylinders being tested. For this purpose, the standard eddy current penetration depth *δ* was used. This parameter defines the depth below the surface of the conductor, at which the current density has fallen to 37%. The *δ* depth ranged from 1.3 mm to 7.3 mm for bronze, from 1.1 mm to 6.0 mm for brass, and from 0.9 mm to 5.1 mm for aluminium.

The error in determining changes in the probe impedance by using the analytical model was negligible. The smallest error values were observed in the reactance calculations. Over the entire frequency range considered, the *dX* coefficient value was less than 2%. The highest absolute values of *dX* were obtained for the aluminium cylinder (1.8%), and the lowest for the ones made of bronze (1.3%) and brass (1.3%). In the calculations of resistance, the error was greater than for the reactance, but in no case exceeded 3%. The *dR* coefficient had the highest absolute value for aluminium (2.9%), lower for brass (2.7%), and the lowest for bronze (2.5%).

As the probe’s operating frequency increases, the values of the impedance components increase. For this reason, a significant influence of disturbance factors can be observed at low frequencies when the impedance values are low. The way the measurement path was configured was to minimise the influence of disturbance factors. To obtain the best possible match, a measuring adapter produced by the manufacturer of LCR metre was used. A solid connection of the power supply cables was achieved by applying clip leads. Before starting the measurements, the metre was calibrated with a precision resistor. The measurements of the impedance components were carried out in multiple mode; therefore, each result was the arithmetic mean of eight measurements. Since the accuracy of determining the probe’s geometric dimensions significantly impacts the calculation results, a digital calliper with an accuracy of ±0.02 mm was used for this purpose. Furthermore, during the probe’s construction, the turns were wound with copper wire in layers to create a regular cylindrical coil. All these measures allowed minimising the influence of undesirable factors upon the values of the measurements and calculations, and due to this, the error in the obtained results did not show sudden changes in values. In the case of resistance, the *dR* coefficient assumed negative values at low frequencies. This means that the resistance changes obtained in the calculations were smaller than the data from the measurements. Along with increasing frequency, the *dR* value was going up, reaching 0% for frequency *f* = 2–3 kHz, and a maximum for *f* = 12–13 kHz. In the case of reactance, the *dX* coefficient value was always negative. The lowest *dX* values were recorded for low frequencies (*f* < 2 kHz).

## 5. Conclusions

The developed mathematical model constitutes a significant step forward in the development of analytical modelling of eddy current problems. The analytical solution for conductive cylinders without defects, known and used so far, has been extended to include the possibility of the occurrence of a hole. This allows the model to be used in defectoscopy for testing various types of cylindrical elements, such as rings, washers or pucks. The input parameters of the model are data entered by a user, such as the radius of the cylinder being tested, its thickness, electrical conductivity, and the radius of the hole. Furthermore, the coil’s geometric dimensions and the number of turns are also specified. Because of this, it is possible to perform calculations for cylinders and coils with various geometric dimensions.

Implementation of the final formulas presented in closed form is possible in any computational package, such as Mathematica or Octave. Calculations of changes in the probe impedance are performed in a short time while maintaining a small error compared to the measurement results. The presented solution to the problem of calculating complex eigenvalues with the employment of the 1D-FEM can be utilised in both existing and many new models. This applies to testing elements containing regions composed of several parts, for example, air–conductor–air. This condition is met by all types of pipes and rings. The extension of the proposed analytical solution will enable the creation of models for more complex geometries, such as coils containing a core or probes composed of multiple coils. It is also being planned to devote future work to replacing the air hole with a conductive material, which will make it possible to detect metal inclusions occurring during manufacturing, and to test double-layer rods.

## Figures and Tables

**Table 1:** Parameters of the eddy current probe used in the experiments.

Parameter	Symbol	Probe
Outer radius	*r*_2_	9.3 mm ± 0.2 mm
Inner radius	*r*_1_	6.5 mm ± 0.2 mm
Height	*z*_2_-*z*_1_	5.0 mm ± 0.1 mm
Number of turns	*N*	1550 ± 2

**Table 2:** Parameters of the conductive cylinders used in the experiments.

Parameter	Bronze	Brass	Aluminium
Electrical conductivity [MS/m]	9.52 ± 0.05	14.24 ± 0.07	19.59 ± 0.10
Thickness [mm]	5.2 ± 0.1	5.1 ± 0.1	5.0 ± 0.1
Outer radius [mm]	25.2 ± 0.1	20.0 ± 0.1	32.5 ± 0.1
Inner radius [mm]	3.0 ± 0.1	2.5 ± 0.1	2.5 ± 0.1

**Table 3:** Value of the complex eigenvalues *p* when *f* = 1 kHz.

No.	Real Part (m^−1^)	Imaginary Part (m^−1^)	No.	Real Part (m^−1^)	Imaginary Part (m^−1^)
1	52.461	1.316	11	405.380	12.815
2	101.934	2.840	12	422.215	159.821
3	151.974	4.395	13	456.622	14.464
4	202.269	6.003	14	500.892	123.492
5	252.757	7.671	15	508.019	16.010
6	288.295	265.875	16	559.503	17.539
7	303.436	9.385	17	588.408	93.920
8	314.060	238.467	18	611.174	18.781
9	354.312	11.113	19	662.697	20.434
10	358.477	200.481	20	680.508	70.492

**Table 4:** Resistance change values obtained for cylinders made of brass, bronze and aluminium.

Conductive Material	Frequency *f* [kHz]	Measurement *R* − *R*_0_ [Ω]	Calculations *R* − *R*_0_ [Ω]	Resistance Error *dR* [%]
Brass	0.5	14.48 ± 0.01	14.14	−2.4
Bronze	0.5	11.82 ± 0.01	11.57	−2.1
Aluminium	0.5	15.01 ± 0.01	14.75	−1.7
Brass	15	295.30 ± 0.15	303.16	2.7
Bronze	15	333.43 ± 0.17	341.61	2.5
Aluminium	15	264.45 ± 0.13	271.47	2.7

**Table 5:** Reactance change values obtained for cylinders made of brass, bronze and aluminium.

Conductive Material	Frequency *f* [kHz]	Measurement *X* − *X*_0_ [Ω]	Calculations *X* − *X*_0_ [Ω]	Reactance error *dX* [%]
Brass	0.5	−9.52 ± 0.01	−9.48	−0.3
Bronze	0.5	−6.24 ± 0.01	−6.17	−1.1
Aluminium	0.5	−13.95 ± 0.01	13.70	−1.8
Brass	15	−1319.19 ± 0.66	−1308.84	−0.8
Bronze	15	−1238.01 ± 0.62	−1225.53	−1.0
Aluminium	15	−1376.66 ± 0.69	−1369.71	−0.5

## Data Availability

The original contributions presented in this study are included in the article. Further inquiries can be directed to the corresponding author.
